# Exploring the potential role of multi-source remote sensing data during different growth stages in crop yield prediction

**DOI:** 10.7717/peerj.21031

**Published:** 2026-04-01

**Authors:** Xingli Gu, Enxiang Xu, Yonggang Chi, Lei Zhou, Qin’ou Liang

**Affiliations:** 1 Zhejiang Normal University College of Geography and Environmental Sciences, Jinhua, China; 2 Zhejiang Normal University Zhejiang Key Laboratory of Digital Intelligence Monitoring and Restoration of Watershed Environment, Jinhua, China

**Keywords:** Grain yield, Solar-Induced Chlorophyll Fluorescence (SIF), Near-Infrared Reflectance of Vegetation (NIRV), Growth period

## Abstract

Accurate prediction of grain yield is essential for enhancing food security, particularly in the context of climate change. Although remote sensing indices have been extensively utilized to monitor vegetation growth and estimate crop yields, there has been limited research comparing their effectiveness for predicting grain yield, especially across different growth stages. This study examined the performance of multi-source indices, such as normalized difference vegetation index (NDVI), near-infrared reflectance of vegetation (NIR_V_), and solar-induced chlorophyll fluorescence (SIF), in predicting grain yield at various growth stages at Shangshan Rice Research Station in Zhejiang Province, China. The results indicated that SIF exhibited the strongest and most consistent correlation with grain yield (*R*^2^ = 0.34 to 0.75), followed by NIR_V_ (*R*^2^ = 0.34 to 0.71). SIF also demonstrated advantages in capturing the dynamic changes of GPP during the reproductive period. During both the vegetative and reproductive stages, leaf area index (LAI) showed significant correlations with NDVI, NIR_V_, and SIF, whereas leaf chlorophyll concentration exhibited comparatively weaker associations with these indicators. These findings provide valuable insights for improving crop yield forecasts using remote sensing, thereby contributing to enhanced agricultural management and food security strategies under climate change.

## Introduction

Accurate forecasts of food production are crucial for ensuring food security, addressing climate change and guiding government policies (Cao et al., 2021; Guo et al., 2024). The total global food demand is expected to increase by 35% to 56% between 2010 and 2050 (Van Dijk et al., 2021). Rice (*Oryza sativa L.*), one of the most extensively cultivated crops worldwide, accounted for 8% of total major crop production in 2021 and plays a vital role in global food security (FAO, 2023). Grain yield estimation has advanced significantly in recent years using remote sensing indices (*e.g.*, vegetation indices) and carbon flux metrics. Gross Primary Productivity (GPP) represents the total carbon assimilation by vegetation through photosynthesis, playing a vital role in crop yield (Lee et al., 2023). Several studies have shown that GPP data can predict corn and soybean yields with superior accuracy (Cheng et al., 2022; Khan, Li & Maimaitijiang, 2024). Furthermore, GPP can be integrated into advanced process models to improve prediction accuracy of grain yields (Lu et al., 2024; Sloat et al., 2021). However, GPP may be influenced by complex processes and factors, such as autotrophic respiration, heterotrophic respiration, and harvest index, during its conversion into final yield (Fernandez-Martinez et al., 2025; Rana et al., 2018). Consequently, developing methods to directly and dynamically predict crop yield remains vital for securing both global and regional food supplies.

Remote sensing has been widely used in predicting crop yield and biomass due to its large-scale, inexpensive, and timely characteristics (Guan et al., 2017). The normalized difference vegetation index (NDVI) is a commonly used parameter that reflects crop growth and nutritional status, allowing for the monitoring of vegetation growth status and cover (Chu et al., 2019). Shammi & Meng (2021) employed 19 growth-related metrics for soybean yield modeling and demonstrated that NDVI-derived indicators exhibited strong predictive performance for estimating soybean yields. Similarly, Johnson (2014) analyzed the relationship between time-series MODIS NDVI data and US county-level corn and soybean yields, and found a significant positive correlation between NDVI and crop yields during the mid-summer period. However, NDVI has limitations, such as saturation occurring in very dense crop canopies and sensitivity to background soil reflectance, which restrict its estimation of biomass and yield (Prabhakara, Dean Hively & McCarty, 2015; Tian et al., 2025). The near-infrared reflectance of vegetation (NIRv) is a novel vegetation index that effectively reduces the influence of background soil reflectance and has been identified as a good proxy for GPP, making it useful for crop yield estimation (Zeng et al., 2021). Peng et al. (2020) demonstrated a significant correlation between the peak value of NIRv and crop yield per unit area for both corn and soybean. However, these indices primarily reflect structural and canopy-level attributes rather than physiological activity. In contrast, solar-induced chlorophyll fluorescence (SIF), a direct byproduct of photosynthetic processes, offers a more physiologically based method for monitoring plant function (Cai et al., 2025; Li, Ding & Xu, 2022b). Wang et al. (2025) integrated satellite SIF with meteorological data and found that SIF explained 78% of county-level corn and soybean yields in the US Midwest (2018–2023). Qiu et al. (2022) demonstrated that GOSIF SIF overcomes the saturation effect of NDVI, improving monitoring accuracy of corn yield losses under drought stress in the Midwest by 23%, significantly outperforming indices such as EVI and NIRv. However, most crop yield estimates in previous studies were based on SIF measurements from satellite-borne sensors. There has been little research on near-surface remote sensing yield estimation, and the results were significantly influenced by spatial resolution (Sloat et al., 2021; Wang et al., 2024).

The appropriate growth stages are crucial in extracting crop growth information and enhancing yield prediction accuracy (Kang et al., 2023). For example, the correlation between NDVI and maize yield was weak after the flowering stage, whereas SIF proved to be more effective in estimating cotton yield during the boll setting and boll opening stages (Kang et al., 2023; Spitko et al., 2016). Similarly, SIF from heading to flowering demonstrated strong yield estimation performance in wheat (Zhu et al., 2023). NIR_V_ from jointing to heading could estimate wheat yield effectively (Li et al., 2022a). In rapeseed yield estimation, NIR_V_ showed a strong correlation with yield during the flowering and budding stages (Fan et al., 2021). In rice, the growth cycle can generally be divided into the seedling, tillering, jointing, heading, flowering, grain filling, and maturity stages, during which changes in canopy structure, pigment content, and photosynthetic activity lead to differences in spectral reflectance and remote sensing responses (Sheng et al., 2022). Selecting the appropriate growth stages is crucial for improving the estimation accuracy of crop yield (Li et al., 2021). However, the optimal growth period for rice yield estimation remains unclear.

The subtropical region is home to about 30% of the global population, a number that continues to grow. However, the per capita arable land in this region is only 40% of that in developed countries (Mao et al., 2023). Additionally, the subtropical region faces numerous challenges, including low agricultural productivity, ecosystem degradation, and climate change (Yuan et al., 2021). Therefore, accurate yield estimation can help relevant departments to plan food production and ensure the stability of the food supply. In this study, we analyzed the relationship between rice grain yield and various indices at different growth stages by utilizing vegetation reflectance spectra and carbon flux parameters. Our objectives are to (1) compare the effectiveness of multi-source indices in predicting rice yield and (2) identify the optimal growth period for estimating rice yield and its influencing factors.

## Materials and methods

### Study site

**Figure 1:**
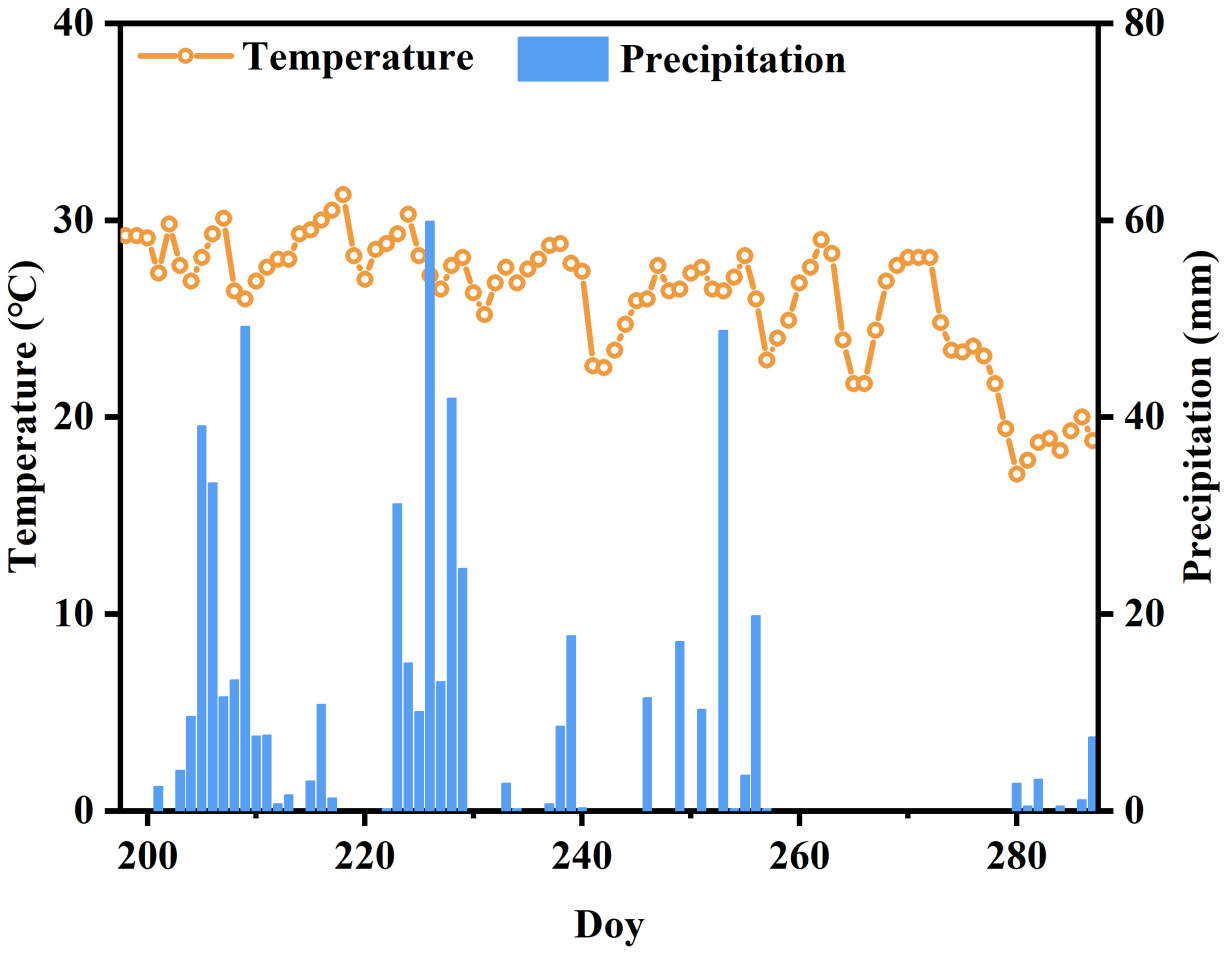
The profile of meteorological variables during the rice growing season.

This study was conducted in 2021 at the Shangshan Rice Research Station (29°27′N, 119°59′E) in Zhejiang Province, China. With an average annual temperature of 16 °C and 1,377 mm of precipitation, the climate in this region was influenced by the East Asian monsoon pattern. The site followed a rapeseed-rice rotation system, and the rice variety used in this study was Yongyou 15. The rice was transplanted on June 19th (DOY 170), with rows spaced 25 cm apart, and harvested on October 24th (DOY 297), resulting in a growing season that stretched for approximately four months. Sixteen plants per square meter was the planting density. The temperature remained relatively stable, fluctuating between approximately 15 °C and 30 °C. Meanwhile, precipitation duration exhibited significant variability (Fig. 1).

### Experimental design

**Figure 2:**
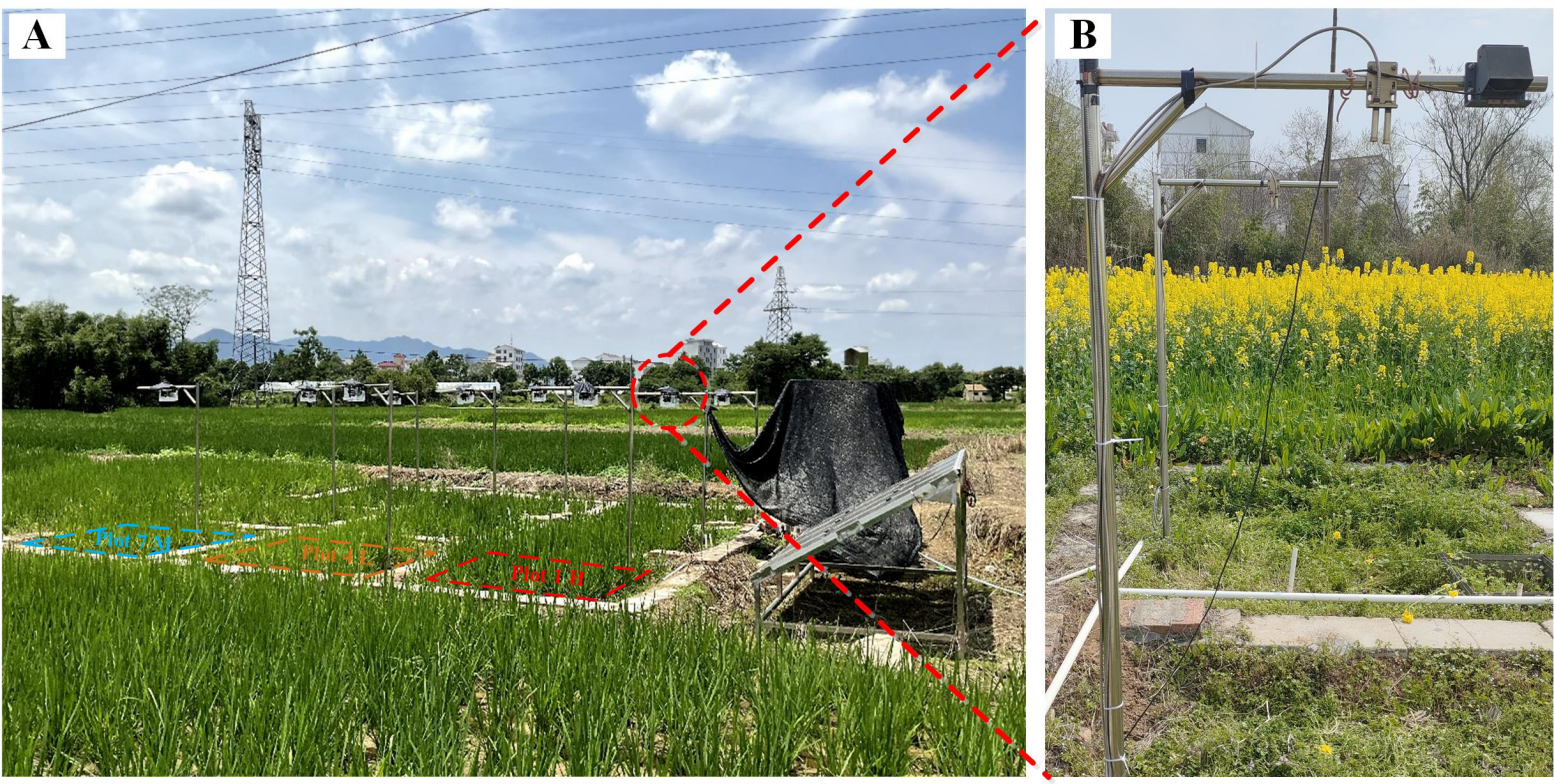
Schematic representation of the field manipulation experiment (A) and the placement of QE Pro fiber during the measurements (B).

The field experiment was conducted using a completely randomized block design with three nitrogen (N) treatment levels: low nitrogen (L, no N application), medium nitrogen (M, 255 kg urea/hm^2^), and high nitrogen (H, 510 kg urea/hm^2^), each with three replicates. In total, nine plots measuring 2  ×   2 m were established. Urea was applied at a ratio of 4:3:3 on DOY 175 (tillering stage), DOY 200 (jointing stage), and DOY 254 (heading stage). Potassium oxide (K_2_O) at 210 kg/ha and phosphorus pentoxide (P_2_O_5_) at 128 kg/ha were applied to all samples. Between DOY 198 and DOY 287, a total of 360 data points were collected at a frequency of every 7 to 10 days (Fig. 2). The flowering stage was identified on DOY 244. Accordingly, the growth period was divided into two distinct phases: the vegetative phase (DOY 198 to DOY 243) and the reproductive phase (DOY 244 to DOY 287).

### Canopy spectral and SIF observation

Throughout the growing season, solar-induced fluorescence (SIF) was monitored using a near-ground automatic multi-angle observation system (Auto-SIF, Bergsun Inc., China). The system was installed 2 m above the canopy. A customized QE Pro spectrometer was utilized, featuring a spectral resolution of 0.14 nm, a spectral sampling interval of 0.07 nm, and a signal-to-noise ratio of 10,000:1 (Fig. 2). Nine sample fibers were connected to the principal fiber *via* an electrical switch, and a standard light source was used for radiometric calibration. Before each measurement in automated observation mode, the instrument modified the integration time according to the intensity of the incident light. Measurements in the “Sandwich” mode were conducted in the following sequence: downward incident radiation, upward canopy radiation, and a second downward incident radiation (Meroni et al., 2008). Daily measurements were conducted every 2-3 s from 08:00 to 18:00 Chinese standard time. The upwelling radiation was measured by the spectrometer through a bare optical fiber with a field of view of 25°. Downwelling incident radiation was measured using a cosine corrector (CC3-3-UV-S, Ocean Optics, Orlando, FL, USA). To minimize the influence of meteorological conditions such as cloud cover, the solar irradiance was averaged over measurements taken before and after the canopy scans. During the measurements of realistic solar radiation and ground reflection spectra, the dark current value was recorded. Spectral data from each quadrat was collected at intervals of 2–3 min. The Sun-Induced Fluorescence (SIF) signal was retrieved using the Spectral Fitting Method (SFM), which described surface reflectance and SIF spectra by employing simple mathematical functions, including polynomial functions and Gaussian functions (Cogliati et al., 2015; Meroni et al., 2010). A quadratic polynomial was used to parameterize the reflectance continuum, and the retrieval was performed over the 758.68–770.37 nm spectral range (Chang et al., 2020).

The ratio of canopy radiance to solar irradiance was calculated for rice yield estimation, utilizing continuous observations from controlled experiments and model simulations (Zhu et al., 2023). The equations of NIR_V_ and NDVI are as follows (Wu et al., 2022b; Zhu et al., 2023):

(1) \begin{eqnarray*}{\mathrm{R}}_{\mathrm{i}}= \frac{{\mathrm{L}}_{\mathrm{i}}}{{\mathrm{I}}_{\mathrm{i}}} \times \pi \end{eqnarray*}

(2) \begin{eqnarray*}{\mathrm{NIR}}_{\mathrm{V}}=\mathrm{NDVI}\times {\mathrm{R}}_{\mathrm{NIR}}= \frac{{\mathrm{R}}_{\mathrm{NIR}}-{\mathrm{R}}_{\mathrm{red}}}{{\mathrm{R}}_{\mathrm{NIR}}+{\mathrm{R}}_{\mathrm{red}}} \times {\mathrm{R}}_{\mathrm{NIR}}\end{eqnarray*}

(3) \begin{eqnarray*}\mathrm{NDVI}= \frac{{\mathrm{R}}_{\mathrm{NIR}}-{\mathrm{R}}_{\mathrm{red}}}{{\mathrm{R}}_{\mathrm{NIR}}+{\mathrm{R}}_{\mathrm{red}}} \end{eqnarray*}

where R_i_ represents the canopy reflectance at I band, R_red_ and R_NIR_ are 680 nm and 770 nm canopy reflectance, respectively.

### GPP calculation

#### Canopy GPP measurements

The canopy CO_2_ fluxes of rice were measured using a Li-6800 infrared gas analyzer (LI-COR Inc., Lincoln, NE, USA), which was equipped with a customized transparent CO_2_ flux chamber that had a base area of 50  ×  50 cm^2^. The chamber was constructed using polyethylene material that transmitted over 90% of photosynthetically active radiation (Günther et al., 2015). Additionally, a custom metallic collar (50  ×  50 cm) was inserted into the soil to permit the connection between the soil and the CO_2_ flux chamber. During the measurements, four small fans installed at the corners of the chamber operated continuously to enhance air mixing within the chamber. Once the CO_2_ concentration began to stabilize, the changes in the chamber’s CO_2_ concentration were recorded continuously at 2-second intervals for 1 min.

The chamber was turned on its side to allow the CO_2_ concentration to recover to ambient levels following the measurement of net ecosystem exchange (NEE). To determine the ecosystem respiration (ER) values, the CO_2_ exchange was then measured once again while the chamber was covered with an opaque fabric. To determine gross primary production (GPP), the absolute values of NEE and ER were added together. In the first five experiments, the chamber height was set at 50 cm, while in the later experiments, the chamber height was increased to 100 cm to accommodate changes in rice growth height. NEE and ER were calculated based on the rate of CO_2_concentration change over time and chamber parameters (Günther et al., 2015).

#### Daily GPP estimated from SCOPE model with input parameters

The SCOPE model is a soil-vegetation-atmosphere-transfer model that simulates the radiative transfer processes of reflected and emitted light (including heat and fluorescence) (Verrelst et al., 2015). This model addresses the limitations of ground-based flux tower data by linking satellite observations with surface processes, thereby enabling better simulation of SIF and GPP at various growth stages of rice (Wang & Xiao, 2021). The input parameters for the SCOPE model include leaf optics, leaf biochemistry, canopy structure, soil properties, fluorescence, and meteorological factors (Table 1). Verrelst et al. (2015) conducted a global sensitivity analysis using the SCOPE model, identifying key variables such as leaf chlorophyll concentration (C_ab_), leaf inclination angle (LIDFa), leaf area index (LAI), and the maximum carboxylation rate (V_cmo_), in addition to various meteorological variables. To simulate the rice canopy SIF and GPP on a daily scale, the following measured values for model input parameters were collected. The simulated values from the SCOPE model fit well with the measured values (Fig. S1).

**Table 1:** Variables and parameters of the SCOPE model for simulating the GPP and LUE of the rice canopies (Van der Tol et al., 2009).

Parameters	Interpretation	Unit	Description	Value
Leaf optical	N	–	Leaf thickness parameters	1.4
	C_ab_	μg/cm^2^	Chlorophyll AB content	Measured
	Cs	–	Scenecent material fraction	0
	Cdm	g/cm^2^	Dry matter content	Measured
Leaf physiology	V_cmo_	μmol/m^2^/s	Maximum carboxylation capacity (at optimum temperature)	Measured
	Rdparam	–	Respiration = Rdparam*Vcmcax	0
Canopy	LAI	M^2^/m^2^	Leaf area index	Calculated
	LIDFa	–	Leaf inclination	−0.35
	LIDFb	–	Variation in leaf inclination	−0.15
Soil	R_ss_	s m^−1^	Soil resistance for evaporation from the pore space	200
Meteorology	R_in_	W m^−2^	Broadband incoming shortwave radiation	Measured
	Ta	°C	Air temperature	Measured

Astronomical radiation (R_a_, MJ m^−2^ d^−1^) and solar incident shortwave radiation (R_s_, W m^−2^) are calculated using site location parameters and sunshine duration. Temperature (Ta,  °C) was the average daily temperature of the site (Allan, Pereira & Smith, 1998). Temperature and sunshine data were obtained from the data center for resources and environmental sciences (https://www.resdc.cn).

(4) \begin{eqnarray*}{\mathrm{R}}_{\mathrm{a}}= \frac{24\times 60}{\pi } {\mathrm{G}}_{\mathrm{sc}}{\mathrm{d}}_{\mathrm{r}} \left( {\omega }_{\mathrm{s}}\sin \nolimits \varphi \sin \nolimits \delta +\cos \nolimits \varphi \cos \nolimits \delta \sin \nolimits {\omega }_{\mathrm{s}} \right) \end{eqnarray*}

(5) \begin{eqnarray*}{\mathrm{R}}_{\mathrm{s}}= \left( {\mathrm{a}}_{\mathrm{s}}+{\mathrm{b}}_{\mathrm{s}} \frac{{\mathrm{n}}_{2}}{\mathrm{N}} \right) {\mathrm{R}}_{\mathrm{a}}\mathrm{ \ast }23.1\end{eqnarray*}

(6) \begin{eqnarray*}\delta =0.409\sin \nolimits ~(2\pi \mathrm{J}/365-1.39)\end{eqnarray*}

(7) \begin{eqnarray*}{\mathrm{d}}_{\mathrm{r}}=1+0.033\cos \nolimits ~(2\pi \mathrm{J}/365)\end{eqnarray*}

(8) \begin{eqnarray*}\mathrm{N}= \frac{24}{\pi } {\omega }_{\mathrm{s}}\end{eqnarray*}

(9) \begin{eqnarray*}\mathrm{J}=\mathrm{int}(275\mathrm{M}/9-30+\mathrm{D})-2\end{eqnarray*}

(10) \begin{eqnarray*}{\omega }_{\mathrm{s}}=\arccos \nolimits ~(-\tan \nolimits \varphi \tan \nolimits \delta )\end{eqnarray*}

(11) \begin{eqnarray*}\varphi =\pi /180\end{eqnarray*}

where G_SC_ is the solar constant of 0.082 (MJ m^−2^ min^−1^), d_r_ is the relative distance between the sun and the earth, *ω*_s_ is the angle of sunset, *φ* is the local latitude (rad), *δ* is the solar declination, a_s_ and b_s_ are the coefficients by which total cosmic radiation reaches the Earth on cloudy and sunny days, respectively, and the recommended values of a_s_ = 0.25 and b_s_ = 0.5 are used here. n_2_/N is the relative sunshine duration, 23.1 is the unit conversion coefficient, D is the number of days, and M is the number of months.

### Calculation of grain yield

In the controlled experiment, each sample was harvested and threshed separately. Then, 100 seeds were randomly selected and weighed to determine the hundred-grain fresh weight. The seeds were heated at 105 °C for 30 min and subsequently dried at 80 °C until they reached a constant weight. Finally, the dried seeds were weighed using a balance with a precision of ten-thousandths of a millimeter to determine the hundred-grain dry weight. The grain yield (kg/m^2^) was calculated by multiplying the dry-to-wet ratio of 100 grains by the total fresh weight of all grains.

(12) \begin{eqnarray*}\text{Grain yield}= \frac{\text{Hundred grains dry weight}}{\text{Hundred grains fresh weight}} \mathrm{ \ast }\text{Fresh weight of all grains}.\end{eqnarray*}

### Calculation of APAR

The formula for absorbed photosynthetically active radiation (APAR) is as follows (Wu et al., 2022a; Zhang et al., 2022). The fraction of absorbed photosynthetically active radiation (fPAR) can be calculated based on the measured NDVI (Miao et al., 2018; Viña & Gitelson, 2005):

(13) \begin{eqnarray*}\mathrm{APAR}=\mathrm{PAR}\times \mathrm{fPAR}={\mathrm{R}}_{\mathrm{s}}\times {\eta }_{\mathrm{Q}}\times \mu \times \mathrm{fPAR}\end{eqnarray*}

(14) \begin{eqnarray*}\mathrm{fPAR}=1.37\times \mathrm{NDVI}-0.17\end{eqnarray*}

where R_S_ is solar short-wave radiation; ŋ_Q_ is the canopy extinction coefficient, set to 0.5 in this study; and μ is the conversion factor between solar irradiance and photosynthesis photon flux density, which is 4.55.

### Canopy structure and vegetation physiological parameters

The LAI data in this study were calculated using the empirical model proposed by Gower, Kucharik & Norman (1999) according to the following relationship:

(15) \begin{eqnarray*}\mathrm{fPAR}=1-{\mathrm{e}}^{-\mathrm{k*LAI}}\end{eqnarray*}

where K is commonly assumed to be 0.5.

Chlorophyll concentration was measured using a spectrophotometric method. Approximately 0.1 g of leaf tissue was sliced into small pieces, then submerged in a solution containing 5 ml of anhydrous ethanol and 5 ml of acetone. The tissue was kept in the dark for 24 h until it was fully decolorized (Arnon, 1949; Wellburn, 1994). The extract was then analyzed spectrophotometrically at wavelengths of 645 and 663 nm (D645, D663).

(16) \begin{eqnarray*}{\mathrm{C}}_{\mathrm{a}}=0.00127\mathrm{ \ast A}663-0.000269\mathrm{ \ast A}645\end{eqnarray*}

(17) \begin{eqnarray*}{\mathrm{C}}_{\mathrm{b}}\mathrm{~ }=0.00229\mathrm{ \ast A}645-0.000468\mathrm{ \ast A}663\end{eqnarray*}

(18) \begin{eqnarray*}{\mathrm{C}}_{\mathrm{ab}}= \left( {\mathrm{C}}_{\mathrm{a}}+{\mathrm{C}}_{\mathrm{b}} \right) \mathrm{ \ast }0.0094\mathrm{ \ast }1{0}^{6}.\end{eqnarray*}

The An-Ci curve of rice leaves was measured using a Li-6800 portable steady-state photosynthesis system (LI-COR Inc., Lincoln, NE, USA), and calculated the maximum carboxylation rate (Sharkey et al., 2007).

### Data analysis

The SCOPE model was used to simulate gross primary productivity (GPP) of rice over 90-day growing season, based on field-measured biophysical and biochemical parameters within sample plots. To investigate the ability of vegetation indices to reflect rice productivity, three remote sensing parameters were extracted: NIR_V_, NDVI, and SIF. Pearson’s correlation analysis was applied to quantify the relationships among these parameters. Furthermore, the relationships between grain yield and remote sensing indices (NIR_V_, NDVI, SIF) were analyzed during the vegetative and reproductive phases, using linear regression models. The coefficient of determination (R^2^) was used to evaluate the explanatory power of each model. To further understand the physiological drivers behind result, we also analyzed the relationships between LAI and C_ab_ with NIR_V_, NDVI, and SIF across both growth stages. Statistical analyses were conducted using MATLAB 2015b (MathWorks, Natick, MA, USA), while data visualization was performed using Origin 2022 (OriginLab, Northampton, MA, USA).

## Results

### Seasonal patterns of multi-source indices

**Figure 3:**
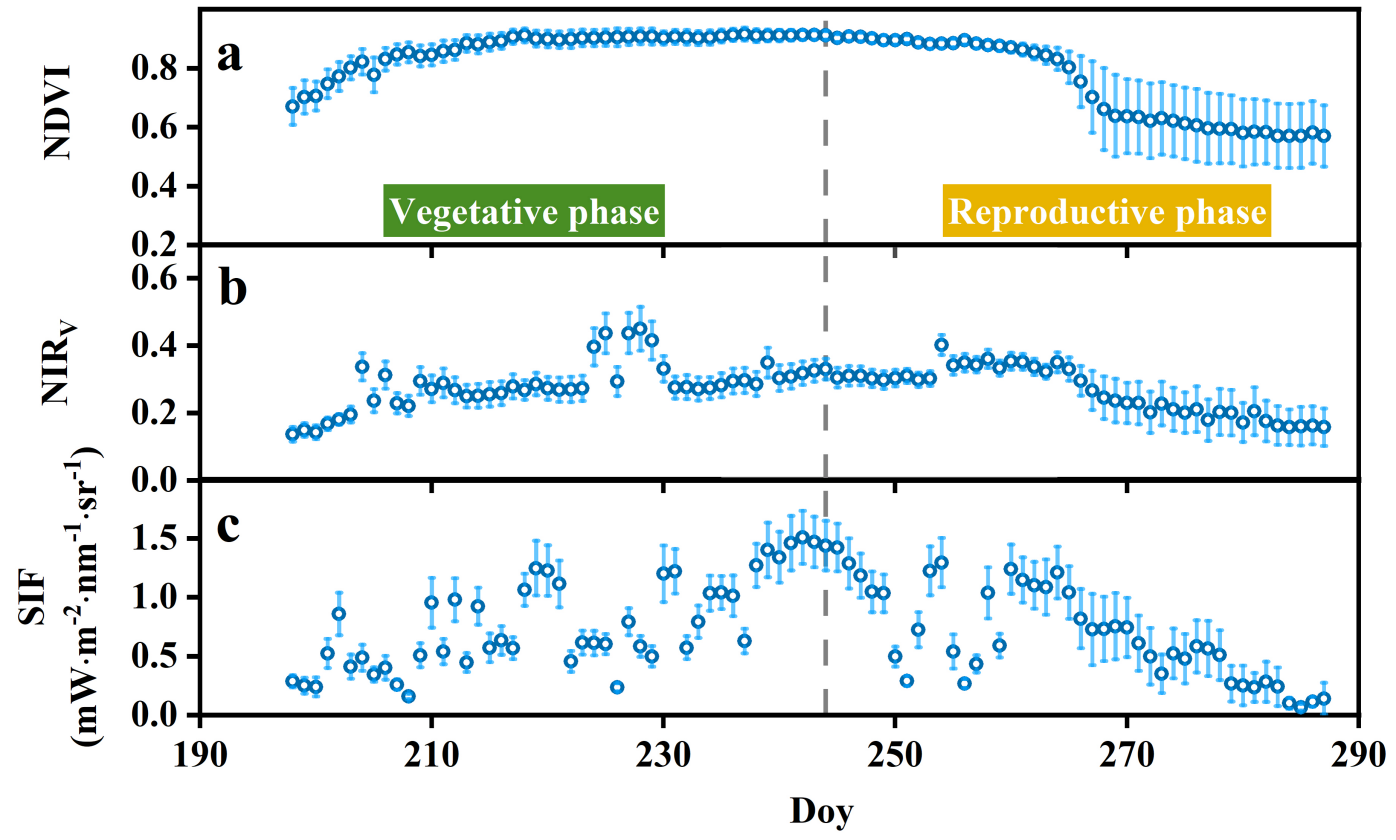
Seasonal variation of daily average NDVI (A), NIR_V_ (B), SIF (C). In the line chart, the points and error bars reflect the mean (±SE) of nine plots. NDVI, normalized difference vegetation index; NIR_*V*_, near-infrared reflectance of vegetation; SIF, solar-induced chlorophyll fluorescence.

Significant correlations were observed among NIR_V_, NDVI, and SIF, with all relationships showing statistical significance (all *p* < 0.05) (Fig. S2). Three indices all exhibited clear seasonal trends. In detail, both NIR_V_ and NDVI showed a gradual increase during the early stages of the growing season, peaking around DOY 230, followed by a subsequent decline (Figs. 3A, 3B). SIF initially displayed a lower value early in the growing season, rising to a peak around DOY 220, and then gradually declining with moderate fluctuations, ranging from −0.046 to 1.875 mW m^−2^ nm^−1^ sr^−1^ (Fig. 3C).

### Relationship between multi-source indices and grain yield

**Figure 4:**
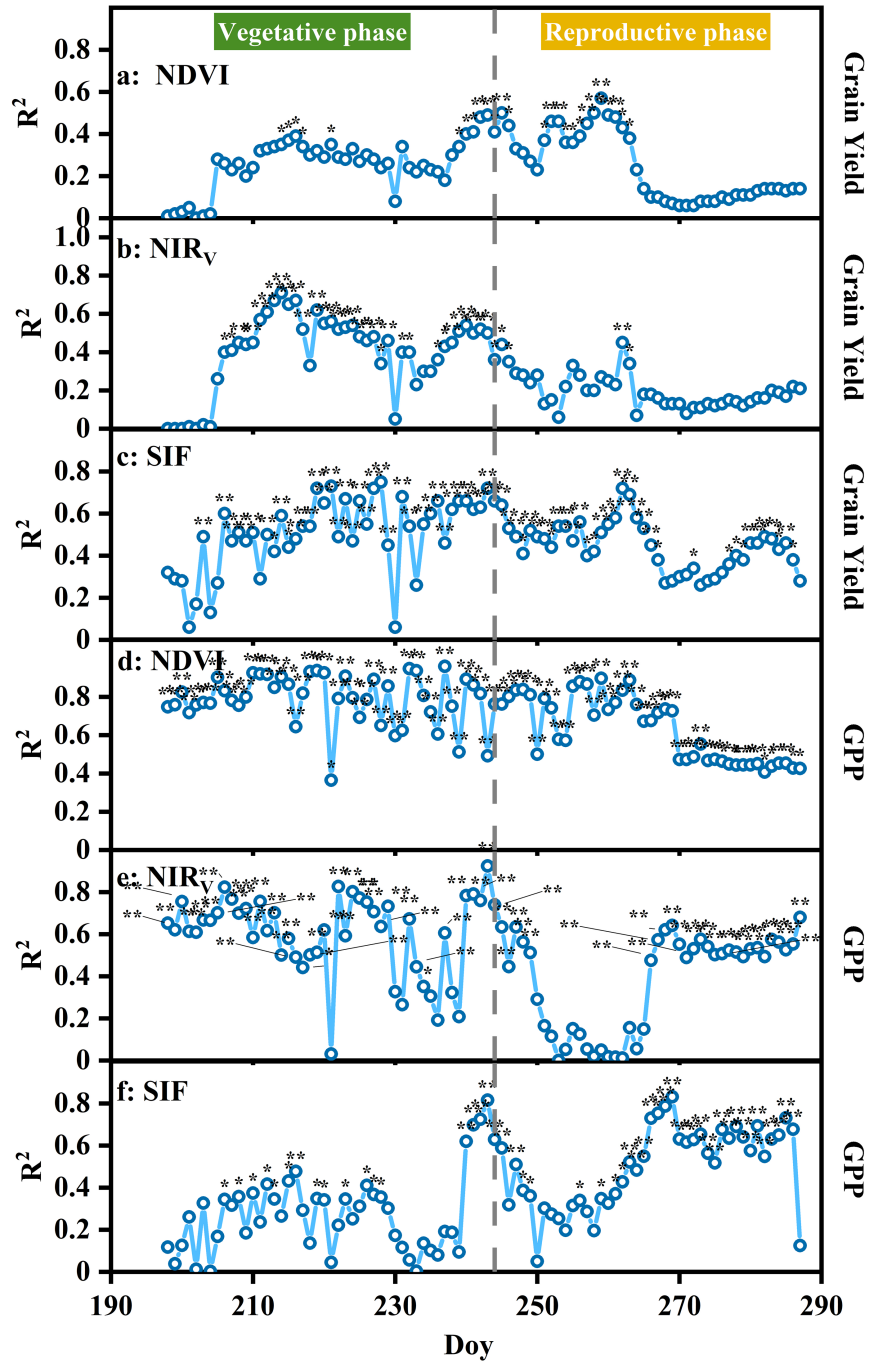
Influence of growth stages on the grain yield prediction (A, B, C) and GPP (D, E, F) by linear regression models developed from NDVI, NIR_*V*_, and SIF. *R*^2^ is the determination coefficient. Different colored backgrounds indicate different growth stages. * *p* < 0.1, ** *p* < 0.05.

SIF and NIR_V_ exhibited the strongest correlations with grain yield across different growth stages (Fig. 4). SIF showed significant correlations throughout almost the entire growth period, peaking during the vegetative phase (*R*^2^ = 0.34 to 0.75, Fig. 4C). NIR_V_ showed significant correlations primarily during the vegetative phases with R^2^ values ranging from 0.34 to 0.71 (Fig. 4A). NDVI also showed significant correlations with grain yield, although the relationship was slightly weaker, with R^2^ values ranging from 0.34 to 0.57 (Fig. 4B). All three indices exhibited significant correlations with GPP throughout the rice growth period (Figs. 4D–4F). NDVI showed the most stable performance, maintaining consistently high correlations with GPP from the vegetative growth stages through maturity, with R^2^ values generally ranging from 0.40 to 0.94 (Fig. 4D). NIR_V_ exhibited similarly strong predictive capacity, particularly during the vegetative phase, with R^2^ values ranging from 0.47 to 0.92 (Fig. 4E). SIF also correlated significantly with GPP across most developmental stages, achieving R^2^ values from 0.34 to 0.83 (Fig. 4F). Overall, these results indicate that while all three indices effectively capture GPP dynamics, NDVI and NIR_V_ are more reliable during vegetative phases, whereas SIF shows higher sensitivity in the reproductive phase.

**Figure 5:**
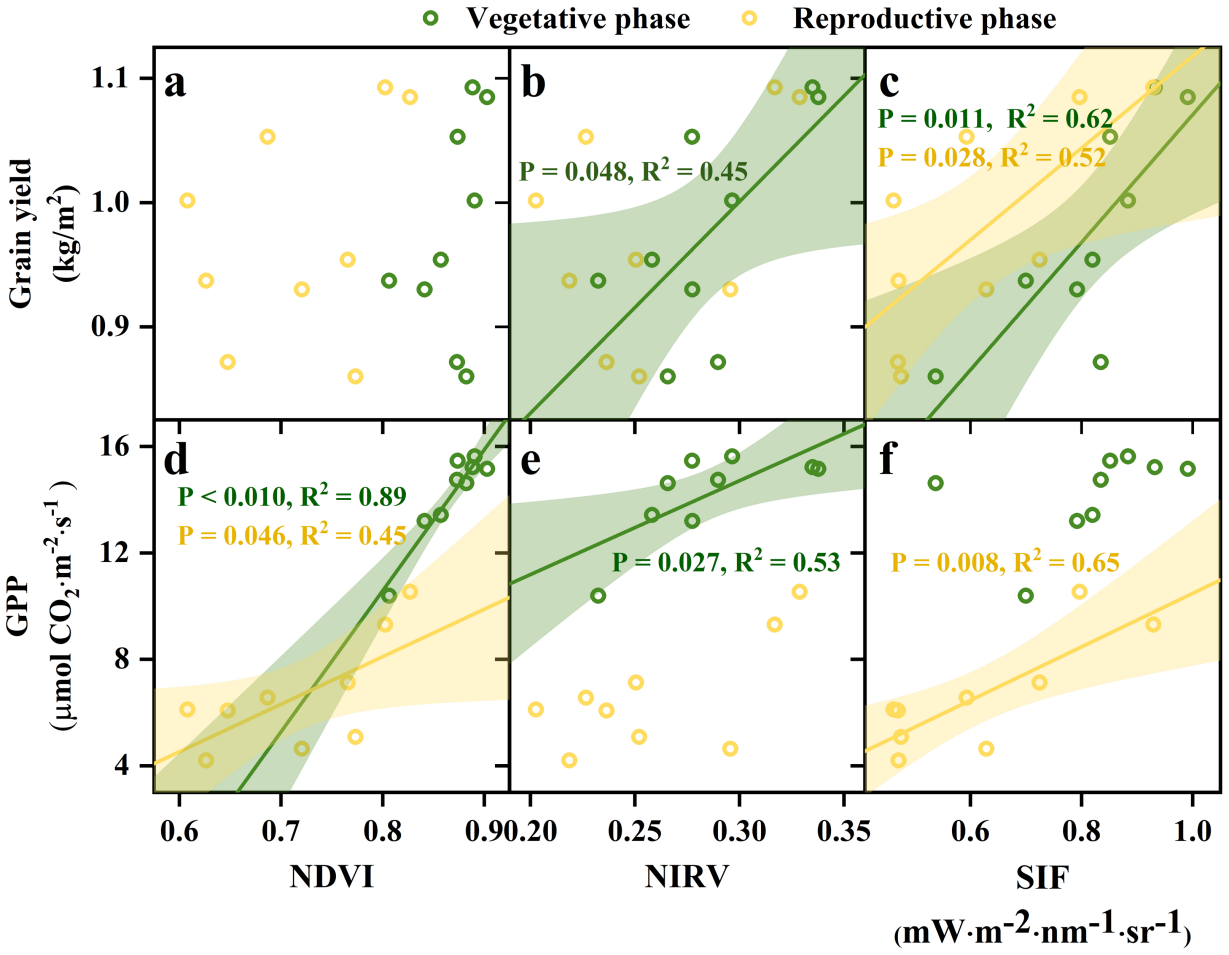
Relationships between grain yield (A, B, C) and GPP (D, E, F) with three indices (NDVI, NIR_*V*_, and SIF) across vegetative and reproductive phases. The panels show the correlation between grain yield with NDVI (A), NIR_V_ (B), SIF (C) across two growth phases: vegetative (green), reproductive (yellow). Solid regression lines and shaded areas represent significant correlations (*P* < 0.05).

The relationship between multi-source indices and grain yield was assessed across two distinct growth phases: the vegetative and reproductive phase (Fig. 5). Among the three evaluated indices, SIF exhibited the strongest correlation with yield, with an R^2^ of 0.62 (*P* = 0.011) in the vegetative phase and R^2^ of 0.52 (*P* = 0.028) in the reproductive phase. NIR_V_ also showed a significant correlation with yield during the vegetative phase (*R*^2^ = 0.45, *P* = 0.048), but no significant relationship was observed during the reproductive phase. In contrast, NDVI did not exhibit any significant correlation with grain yield in either growth stage. In addition to grain yield, we further assessed the relationships of NDVI, NIR_V_, and SIF with GPP across the two phases (Figs. 5D–5F). NDVI exhibited the strongest predictive capability for GPP, yielding an R^2^ of 0.89 (*P* < 0.010) during the vegetative stage and a moderate but significant correlation during the reproductive stage (*R*^2^ = 0.45, *P* = 0.046). NIR_V_ also showed a significant association with GPP during vegetative growth (*R*^2^ = 0.53, *P* = 0.027), although its predictive strength declined in the reproductive phase. In contrast, SIF demonstrated its highest sensitivity during the reproductive stage, exhibiting a robust correlation with GPP (*R*^2^ = 0.65, *P* = 0.008).

### Relationship between LAI and C_ab_ with multi-source indices

**Figure 6:**
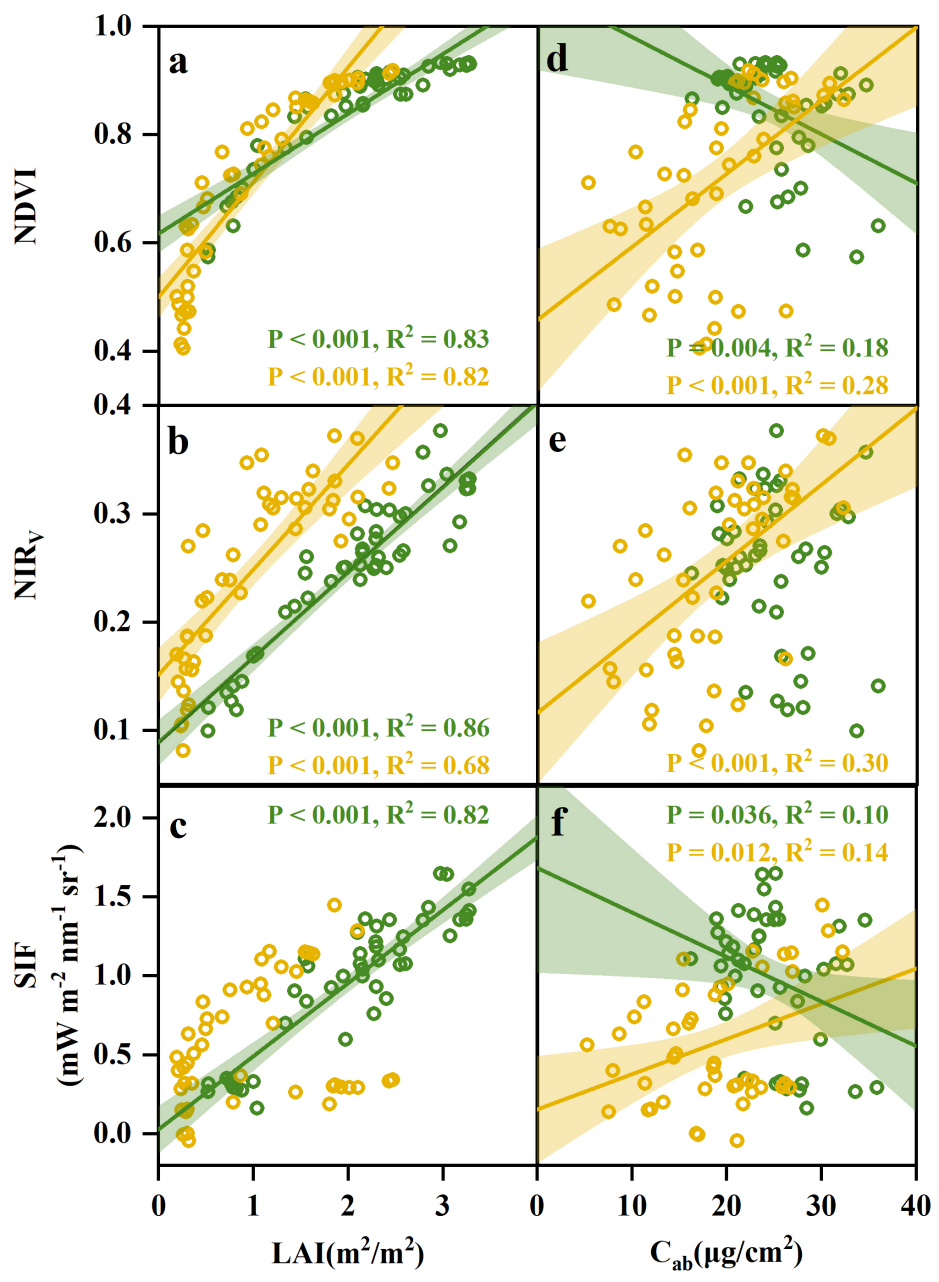
Relationships between LAI (A, B, C) and C_ab_. (D, E, F) with three indices (NDVI, NIR_*V*_, and SIF) across vegetative and reproductive phases. Green and yellow dots indicate data from the vegetative and reproductive phases, respectively. Shaded regions represent 95% confidence intervals for significant regressions (*P* < 0.05). LAI, leaf area index; *C*_*ab*_, leaf chlorophyll concentration.

Our results demonstrated that LAI had a stronger influence than C_ab_ on all three indices (Fig. 6). NIR_V_ showed extremely strong positive correlations with LAI, with *R*^2^ = 0.86 (*P* < 0.001) in the vegetative phase and *R*^2^ = 0.68 (*P* < 0.001) in the reproductive phase (Fig. 6A). Similar patterns were observed for NDVI and SIF, which also showed high correlations with LAI in the vegetative phase (NDVI: *R*^2^ = 0.83, SIF: *R*^2^ = 0.82). The relationships between C_ab_ and the vegetation indices were weaker and more variable (Figs. 6D–6F). C_ab_ and NIR_V_ were correlated during the reproductive phase (*R*^2^ = 0.30, *P* < 0.001), while C_ab_ and NDVI exhibited correlations in the vegetative phase (*R*^2^ = 0.18) and reproductive phase (*R*^2^ = 0.28). C_ab_ and SIF showed the weakest correlations, with R^2^ values of 0.10 (*P* = 0.036) and 0.14 (*P* = 0.012) in the vegetative and reproductive phases, respectively.

## Discussion

### SIF and NIR_**V**_ are superior predictors of rice yields

Our study demonstrated that SIF provided robust estimation of rice grain yield by comparing the effectiveness of multi-source indices in subtropical region of China (Fig. 5). This was in agreement with the results of wheat yield estimation using multiple machine learning models in the Gangetic Plains of India (Liu et al., 2022). Similarly, a study in the United States demonstrated that SIF accounted for the variability in wheat yield (Joshi et al., 2023). The superior performance of SIF in yield estimation was attributed to its ability to accurately capture photosynthesis in crops (Wang et al., 2023). Photosynthesis influences crop yield directly, as over 90% of a crop’s dry matter is produced through this process (Wang et al., 2022). Meanwhile, SIF demonstrated superior performance compared to other vegetation indices for yield estimation because SIF was sensitive to canopy structure and vegetation physiology (Song et al., 2018; Wang et al., 2022). The relationship between SIF and LAI was significant in the vegetative phase (Fig. 6). SIF was found to be closely correlated with LAI and chlorophyll content in a study on the connection between vegetation canopy senescence and wheat yield (Zhu et al., 2023). Therefore, SIF should be prioritized in exploring crop yield at the regional scale.

NIR_V_ was second to SIF in predicting crop yield. Similar to SIF, NIR_V_ also detects photosynthesis more efficiently than NDVI (Zhu et al., 2023). NIR_V_ indicates the proportion of NIR light reflected from the vegetative canopy, allowing for the separation of vegetation signals from the soil background, distinguishing photosynthesis distribution with canopy depth, and capturing more valuable information for productivity modeling (Li, Ding & Xu, 2022b). Fan et al. (2021) found the great potential of the NIR_V_ in the flowering stage to provide the accurate early prediction of the rapeseed yield. Similarly, Peng et al. (2020) demonstrated that NIR_V_ had the best overall performance in predicting maize and soybean yield based on the assessed benefits of using satellite-based SIF products in the US Midwest. Building on this, Li et al. (2022a) reported that an NIR_V_-based random forest model slightly outperformed NDVI- and EVI-based models in predicting wheat yield in China. The relationship between NIR_V_ and LAI was significant in the vegetative and reproductive phases (Fig. 6). Our study emphasizes that seasonal dynamics of NIR_V_ are useful for tracking crop yield in subtropical croplands.

### SIF is a superior predictor for estimating GPP during the reproductive phase

We found that NDVI and NIR_V_ exhibited stronger correlations with GPP during the vegetative phase, whereas SIF showed a clear advantage in capturing GPP dynamics during the reproductive phase (Fig. 5). This phase-dependent performance difference can be attributed to shifts in rice carbon allocation strategies and the decoupling between canopy structure and physiological function across developmental stages (Ding et al., 2026). During the vegetative phase, rice growth is dominated by rapid leaf expansion and canopy structure establishment (Zhong et al., 2024). During this period, LAI and chlorophyll content increase rapidly, enhancing light interception and carbon assimilation for biomass accumulation (Fig. S3). Vegetation indices that are sensitive to canopy structure and chlorophyll content effectively reflect variations in GPP (Dechant et al., 2020). During the early reproductive phase, photosynthetic activity often declines due to the effects of climatic constraints and physiological regulation associated with carbon reallocation, while canopy structure and chlorophyll content remain relatively stable (Zhang et al., 2022). As a result, NDVI and NIR_V_ often remain stable prior to visible foliar yellowing, leading to a lagged response relative to the decline in actual photosynthetic capacity (Jeong et al., 2017). In contrast, SIF is directly linked to photosynthetic regulation and energy partitioning within the photosystems (Guanter et al., 2014; Magney et al., 2019). Similar phase-dependent patterns have been reported in winter wheat, where SIF demonstrated greater robustness across temporal scales and superior performance during the reproductive phase, when NDVI tends to saturate under dense canopies (Peng et al., 2020). Li et al. (2020) further showed that the slope of the SIF–GPP relationship increased throughout the growing season, with the strongest correlation occurring during the reproductive stage.

It should be noted that the measurements in this study did not fully cover the senescence period and relied on linear regression models, which may not adequately capture complex, nonlinear interactions among growth dynamics, physiological processes, and environmental factors. Previous studies have shown that during senescence, increasing non-linearity may lead linear models to underestimate the SIF–GPP relationship (Liu et al., 2020). Emerging studies increasingly employ machine learning to integrate multi-source remote sensing data across phenological stages for dynamic yield prediction (Croci et al., 2022). Future work should account for nonlinear processes to further improve GPP and yield estimation. In summary, while NDVI and NIR_V_ reliably track GPP during canopy expansion, they exhibit distinct advantages for estimating GPP during the reproductive phase.

## Conclusion

In this study, seasonal variations of multi-source indices, including NIR_V_, NDVI, and SIF were observed throughout the growing season in subtropical rice paddies. We compared the effectiveness of vegetation reflectance spectra and carbon flux parameters in estimating rice grain yield. Our results indicated that both NIR_V_ and SIF exhibited strong correlations with rice grain yield, surpassing the performance of other indices such as NDVI. NDVI and NIR_V_ are reliable indicators of GPP during canopy expansion, SIF holds irreplaceable advantages during the reproductive growth stage. This finding provides valuable insights into the yield prediction in subtropical regions.

##  Supplemental Information

10.7717/peerj.21031/supp-1 Supplemental Information 1 Experimental results Results of correlation analysis between multi-source indices and production.

10.7717/peerj.21031/supp-2 Supplemental Information 2 Supplementary Figures

## Additional Information and Declarations

### Data Availability

The following information was supplied regarding data availability: The raw measurements are available in the Supplemental File.
