# Supplementary material for: Exploring the potential role of multi-source remote sensing data during different growth stages in crop yield prediction
Source: PeerJ. 2026 Apr 1;14:e21031. doi: 10.7717/peerj.21031 (PMC13050220; doi:10.7717/peerj.21031)
Supplement: Supplemental Information 2 [file peerj-14-21031-s002.doc]

*PeerJ*

Supporting Information for

**Exploring the potential role of multi-source remote sensing data during different growth stages in crop yield prediction**

Xingli Gu^1^, Enxiang Xu^1^, Yonggang Chi^1,2^*, Lei Zhou^1,2^, Qin’ou Liang^1,2^*

^1^College of Geography and Environmental Sciences, Zhejiang Normal University, Jinhua 321004, China

^2^Zhejiang Key Laboratory of Digital Intelligence Monitoring and Restoration of Watershed Environment, Zhejiang Normal University, Jinhua, Zhejiang, 321004, China

**Contents of this file**

Figures S1 to S3

**Introduction**

This supporting information file contains supplementary figures, all of which are referred to in the main text.


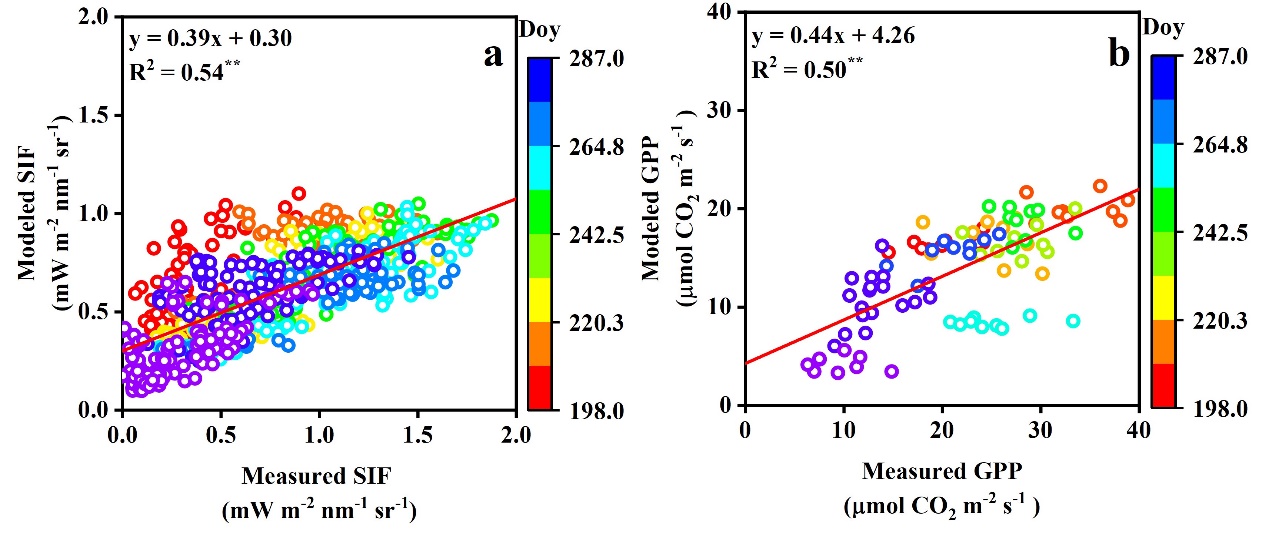


**Fig. S1** Relationship between the measured data and the simulation results of the SCOPE model. *: p<0.05, **: p<0.01.


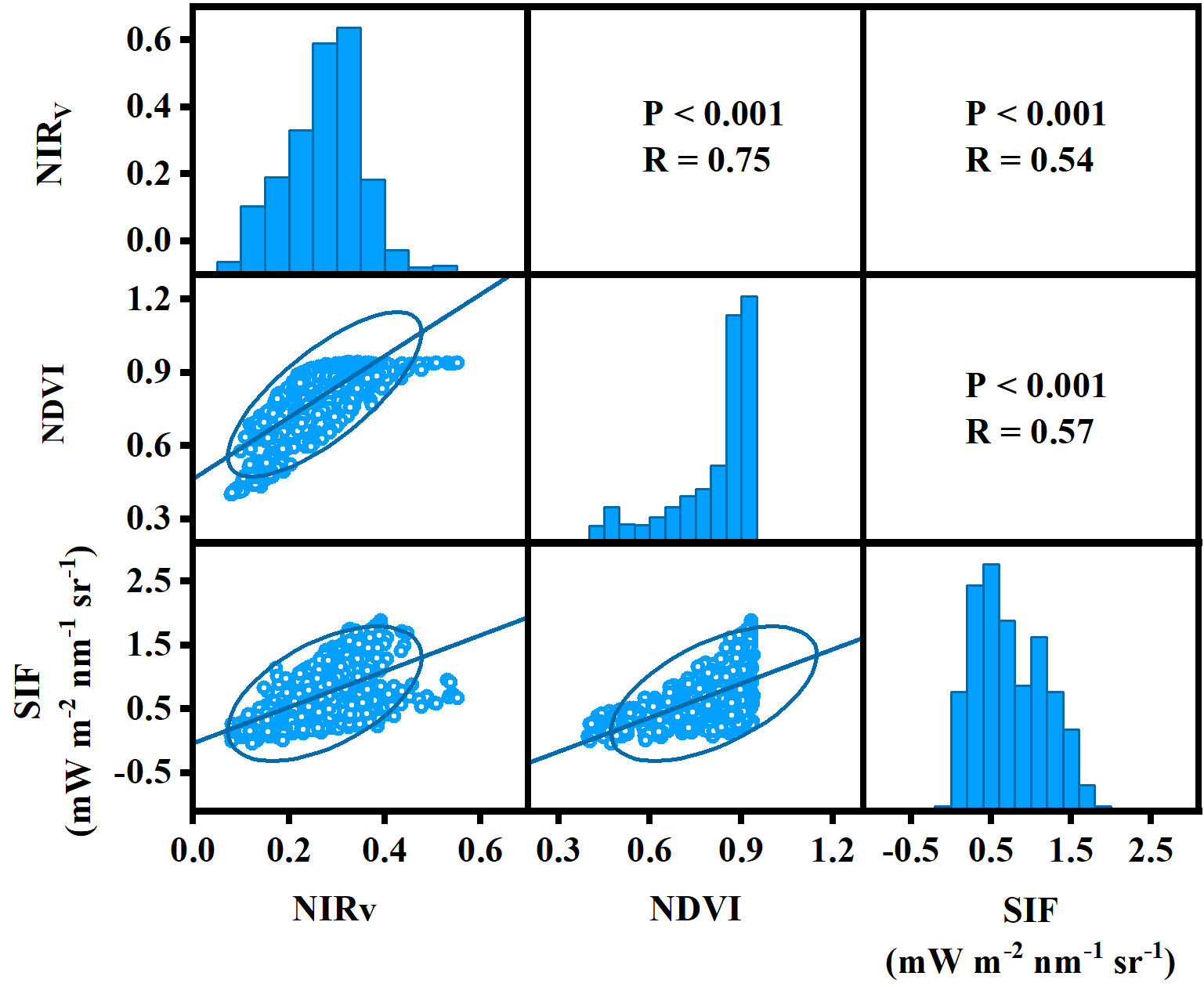


**Fig. S2** Relationship between multi-source indices. P is the Pearson correlation significance level, and R is the Pearson correlation coefficient. The ellipse is a 95% confidence ellipse and the diagonal bar plot is a histogram of the distribution of the data. NIR_V_, near-infrared reflectance of vegetation; NDVI, normalized difference vegetation index; SIF, solar-induced chlorophyll fluorescence.


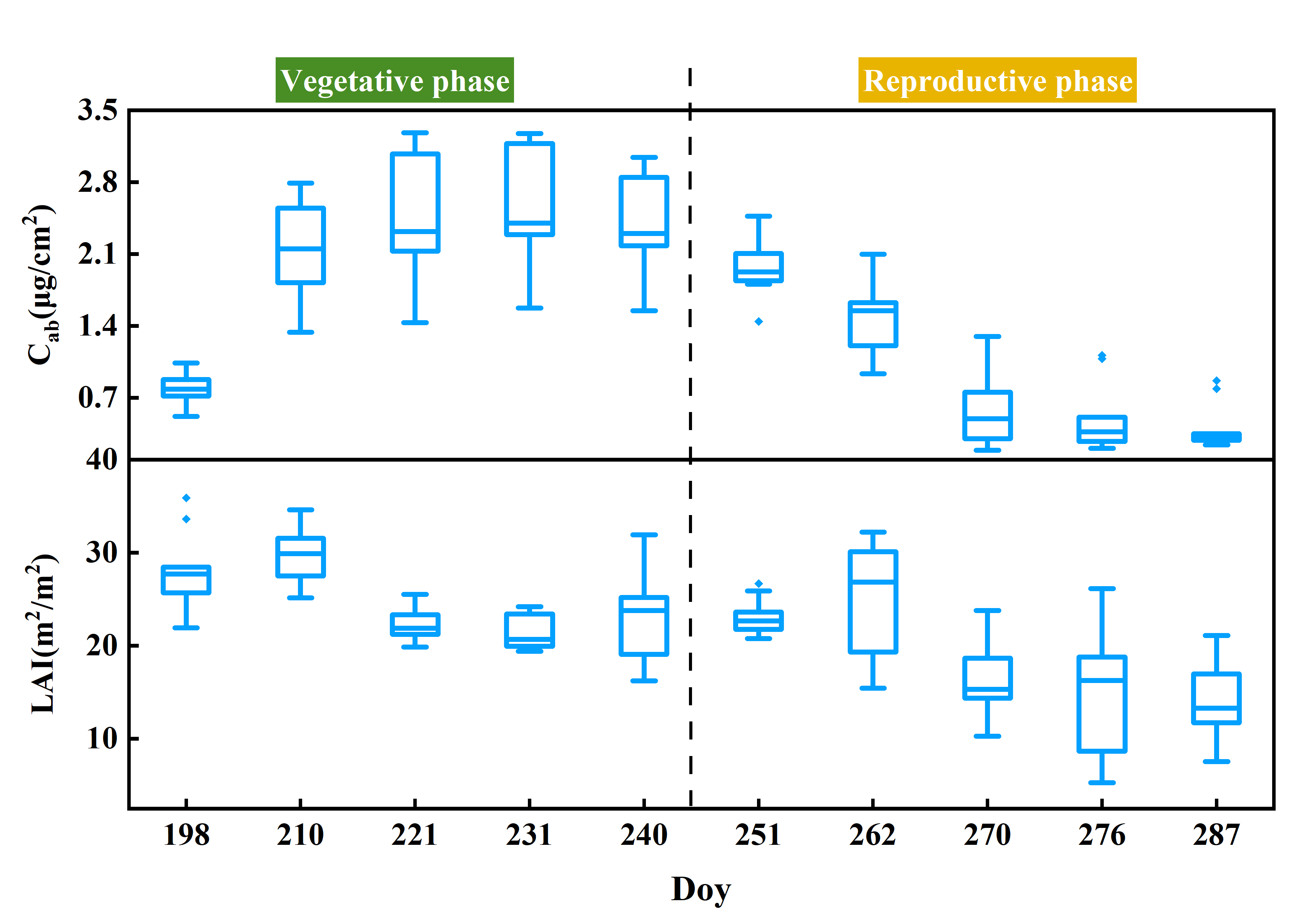


**Fig. S3** Seasonal dynamics of Cab and LAI during the rice growing season. The vegetative and reproductive phases are demarcated by a vertical dashed line at DOY 244.


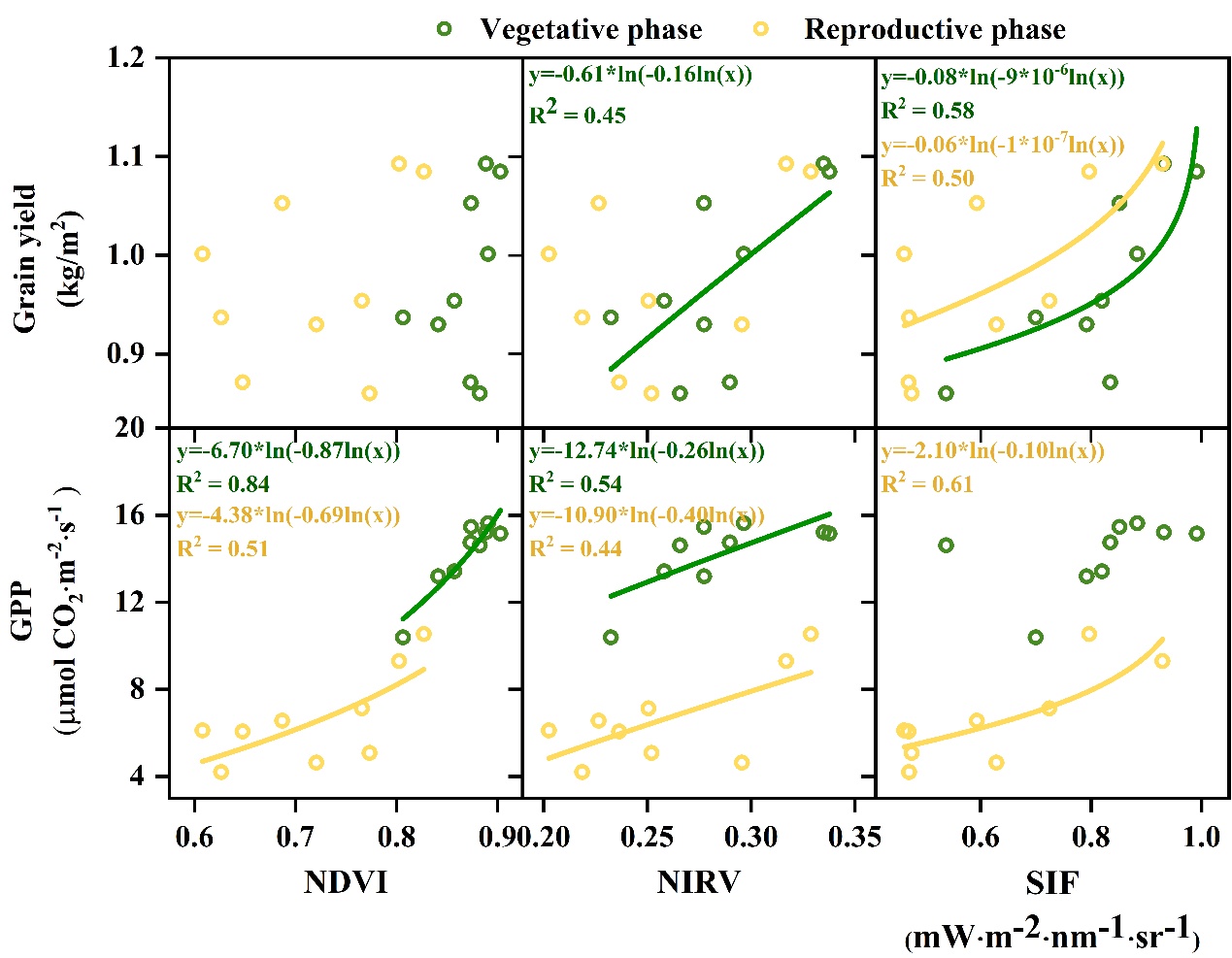


**Fig. S4** Non-linear relationships between grain yield (a, b, c) and GPP (d, e, f) with NDVI, NIRV, and SIF across vegetative and reproductive phases. Green and yellow dots indicate observations from the vegetative and reproductive phases, respectively.
